# Supplementary material for: Bayesian refinement of protein structures and ensembles against SAXS data using molecular dynamics
Source: PLoS Comput Biol. 2017 Oct 18;13(10):e1005800. doi: 10.1371/journal.pcbi.1005800 (PMC5662244; doi:10.1371/journal.pcbi.1005800)
Supplement: S1 Archive — (BZ2) [file pcbi.1005800.s011.tar.bz2 › gromacs_4.62_bayesian_saxs_refi/share/html/online/g_angle.html]

g\_angle


| g\_angle Main Table of Contents |

**VERSION 4.6  
Sat 19 Jan 2013**

---

### Description

g\_angle computes the angle distribution for a number of angles
or dihedrals.

With option -ov, you can plot the average angle of
a group of angles as a function of time. With the -all option,
the first graph is the average and the rest are the individual angles.

With the -of option, g\_angle also calculates the fraction of trans
dihedrals (only for dihedrals) as function of time, but this is
probably only fun for a select few.

With option -oc, a dihedral correlation function is calculated.

It should be noted that the index file must contain
atom triplets for angles or atom quadruplets for dihedrals.
If this is not the case, the program will crash.

With option -or, a trajectory file is dumped containing cos and
sin of selected dihedral angles, which subsequently can be used as
input for a principal components analysis using g\_covar.

Option -ot plots when transitions occur between
dihedral rotamers of multiplicity 3 and -oh
records a histogram of the times between such transitions,
assuming the input trajectory frames are equally spaced in time.

### Files

| option | filename | type | description |
| --- | --- | --- | --- |
| **-f** | traj.xtc | Input | Trajectory: xtc trr trj gro g96 pdb cpt |
| **-n** | angle.ndx | Input | Index file |
| **-od** | angdist.xvg | Output | xvgr/xmgr file |
| **-ov** | angaver.xvg | Output, Opt. | xvgr/xmgr file |
| **-of** | dihfrac.xvg | Output, Opt. | xvgr/xmgr file |
| **-ot** | dihtrans.xvg | Output, Opt. | xvgr/xmgr file |
| **-oh** | trhisto.xvg | Output, Opt. | xvgr/xmgr file |
| **-oc** | dihcorr.xvg | Output, Opt. | xvgr/xmgr file |
| **-or** | traj.trr | Output, Opt. | Trajectory in portable xdr format |

### Other options

| option | type | default | description |
| --- | --- | --- | --- |
| **-[no]h** | bool | no | Print help info and quit || **-[no]version** | bool | no | Print version info and quit || **-nice** | int | 19 | Set the nicelevel || **-b** | time | 0 | First frame (ps) to read from trajectory || **-e** | time | 0 | Last frame (ps) to read from trajectory || **-dt** | time | 0 | Only use frame when t MOD dt = first time (ps) || **-[no]w** | bool | no | View output .xvg, .xpm, .eps and .pdb files || **-xvg** | enum | xmgrace | xvg plot formatting: xmgrace, xmgr or none || **-type** | enum | angle | Type of angle to analyse: angle, dihedral, improper or ryckaert-bellemans || **-[no]all** | bool | no | Plot all angles separately in the averages file, in the order of appearance in the index file. || **-binwidth** | real | 1 | binwidth (degrees) for calculating the distribution || **-[no]periodic** | bool | yes | Print dihedral angles modulo 360 degrees || **-[no]chandler** | bool | no | Use Chandler correlation function (N[trans] = 1, N[gauche] = 0) rather than cosine correlation function. Trans is defined as phi < -60 or phi > 60. || **-[no]avercorr** | bool | no | Average the correlation functions for the individual angles/dihedrals || **-acflen** | int | -1 | Length of the ACF, default is half the number of frames || **-[no]normalize** | bool | yes | Normalize ACF || **-P** | enum | 0 | Order of Legendre polynomial for ACF (0 indicates none): 0, 1, 2 or 3 || **-fitfn** | enum | none | Fit function: none, exp, aexp, exp\_exp, vac, exp5, exp7, exp9 or erffit || **-ncskip** | int | 0 | Skip this many points in the output file of correlation functions || **-beginfit** | real | 0 | Time where to begin the exponential fit of the correlation function || **-endfit** | real | -1 | Time where to end the exponential fit of the correlation function, -1 is until the end |

### Known problems

- Counting transitions only works for dihedrals with multiplicity 3

---

http://www.gromacs.org  
gromacs@gromacs.org
